# An improved deep forest model for prediction of e-commerce consumers’ repurchase behavior

**DOI:** 10.1371/journal.pone.0255906

**Published:** 2021-09-20

**Authors:** Weiwei Zhang, Mingyan Wang

**Affiliations:** School of Management, Shanghai University of Engineering Science, Songjiang, Shanghai, China; Shandong University of Science and Technology, CHINA

## Abstract

As the Internet retail industry continues to rise, more and more consumers choose to shop online, especially Chinese consumers. Using consumer behavior data left on the Internet to predict repurchase behavior is of great significance for companies to achieve precision marketing. This paper proposes an improved deep forest model, and the interactive behavior characteristics of users and goods are added into the original feature model to predict the repurchase behavior of e-commerce consumers. Based on the Alibaba mobile e-commerce platform data set, first construct a feature engineering that includes user characteristics, product characteristics, and interactive behavior characteristics. And then use our proposed model to make predictions. Experiments show that the model’s overall performance with increased interactive behavior features is better and has higher accuracy. Compared with the existing prediction models, the improved deep forest model has certain advantages, which not only improves the prediction accuracy but also reduces the cost of training time.

## 1. Introduction

**Fig 1:**
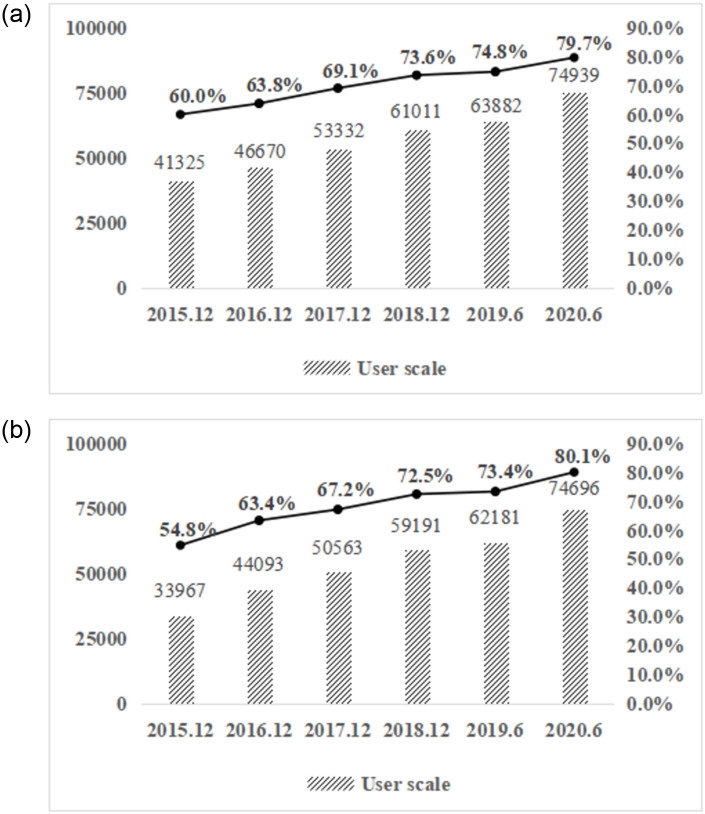
2015.12-2020.3 scale of internet users in China. (a) 2015.12-2020.3 online shopping user scale and utilization rate. (b) 2015.12-2020.3 mobile online shopping user scale and utilization rate.

With the advent of the big data era, available information grows explosively. In 2011, digital information capacity increased nine times in just five years. By 2020, the global information volume has reached 35 trillion gigabytes [1]. Most of the data come from historical records left by users on the Internet, such as web search [2], clicks [3], etc. As shown in Fig 1, by June 2020, the number of online shopping users in China has reached 749 million [4]. Compared with 2018, the number increased by nearly 139 million in 2020, accounting for 79.7 percent of the Internet users. The scale of mobile online shopping has reached 747 million. Compared with 2018, 2020 increased by 156 million, accounting for 80.1 percent of mobile Internet users. Victor Meyer Schönberg, a well-known data scientist, once said that “the core of big data is prediction” [5]. By mining big data’s hidden laws, meaningless data can realize value [6, 7]. In e-commerce, enterprises can predict users’ needs and preferences based on the consumers’ behavioral data [8].

In recent years, more and more scholars have focused on the prediction of consumer behavior. Research on the consumer repurchase behavior prediction has begun with the planned behavior theory. Most of the studies focus on the discussion of purchase intention [9–11] and influencing factors [12–15]. Later, the methods of mathematical statistics were introduced to simplify the behavior prediction into a two-category problem [16, 17]. KNN is one of the simplest classification methods, which is widely used in vehicle sales forecast [18], health monitoring [19], housing price forecast [20], and other fields. Similar to KNN, SVM is also a popular classification method, which is based on the structural risk minimization (SRM) principle of statistical learning theory and has excellent generalization performance [21–23]. However, KNN and SVM are more suitable for simple linear data. When the data is complex and has a non-linear structure, it is challenging to obtain ideal prediction results [24]. Random Forest has excellent advantages in non-linear problems [25].

Nevertheless, as the data becomes larger and larger, the accuracy of the forecast results gradually declines. Oscar Claveria found that the Artificial Neural Network model ANN has good prediction results for non-linear data when studying the number of tourist forecasts [26]. Dennis Koehn uses clickstream data to predict online shopping behavior. After comparing multiple classifiers, it was found that the model combining RNN and traditional classifiers was superior to those considered in other studies. The neural network-based model has an excellent predictive effect [27, 28], but also requires a large number of data sets for training and a lot of hyper parameters [29], which is not conducive to practical operation.

The prediction of customers’ behavior can increase loyal users’ viscosity and achieve precision marketing. Based on this background, we proposed an improved deep forest model to predict repeat purchase behavior of e-commerce consumers. Deep forest, also known as multi-granularity cascading forest (gcForest), is a new tree-based integration method proposed in 2017. In Professor Zhou’s experiment, the deep forest has higher accuracy than traditional machine learning methods [30]. When analyzing and predicting mineral prospects, Chica-Olmo also discovered that the tree-based model has higher robustness and better ROC value [31]. The tree-based model has two advantages [32–34]. First, it can convert features into binary values, an efficient and easy way to reduce dimension. Simultaneously, it does not need to model these manifolds’ non-linear geometry like the manifold learning method. Second, it can eliminate the non-linearity in features and improve the linear classifiers, while linear transformations cannot. Moreover, learning deep neural network models from large samples is time-consuming. In theory, the prediction model based on gcForest has the characteristics of high accuracy, high robustness, and low training time.

Previous studies on consumer behavior focused on analyzing influencing factors of repurchase behavior [9–12, 15], but this paper focuses on predicting consumer repurchase behavior. In consumer behavior prediction experiments, previous studies have mostly considered user factors and commodity factors [35, 36]. In our experiment, we added the dimension of user-commodity interaction behavior to feature engineering. Moreover, we improved the cascade layer in the deep forest model, and improved the random forest layer into two layers of Extreme random Trees (ET) and two layers of eXtreme Gradient Boosting (XGBoost). This paper is based on the large amount of behavioral data left by consumers on the Internet to predict whether consumers will buy this product repeatedly within one month. Experimental results show that the developed gcForest-based model is more successful than traditional machine learning models in predicting consumer repeat purchase behavior.

The remaining part of this paper proceeds as follows. Section 2 briefly describes the deep forest model and its cascading layers, including the ET model and XGBoost model. Section 3 describes the experimental background in detail. In section 4, the real data set results are analyzed and compared with other prediction models’ results. In Section 5, we discuss and summarize this experiment.

## 2. Methods

The framework of the proposed method is shown in this section, which consists of three parts. The first part introduces the deep forest model as the basic framework, an ensemble learning method that imitates the neural network structure. The second part introduces the extreme random tree model and XGBoost model as cascading forest layers. The third part introduces the improved deep forest model.

### 2.1 Prediction framework of deep forest

The deep forest is a supervised ensemble algorithm with excellent generalization performance, proposed by Zhou and Feng in 2017 [30]. The deep forest model’s depth is adaptive, and diversity can be further enhanced by automatically adjusting the depth. Therefore, even with the input of high-dimensional data, the deep forest model can obtain relatively excellent results, which are comparable to the deep neural networks to some extent. Moreover, the deep forest model has far fewer hyperparameters than the deep neural network. Furthermore, the parameter settings are relatively robust [33], and desirable results can be obtained using default parameters for different experiments and data sets.

**Fig 2:**
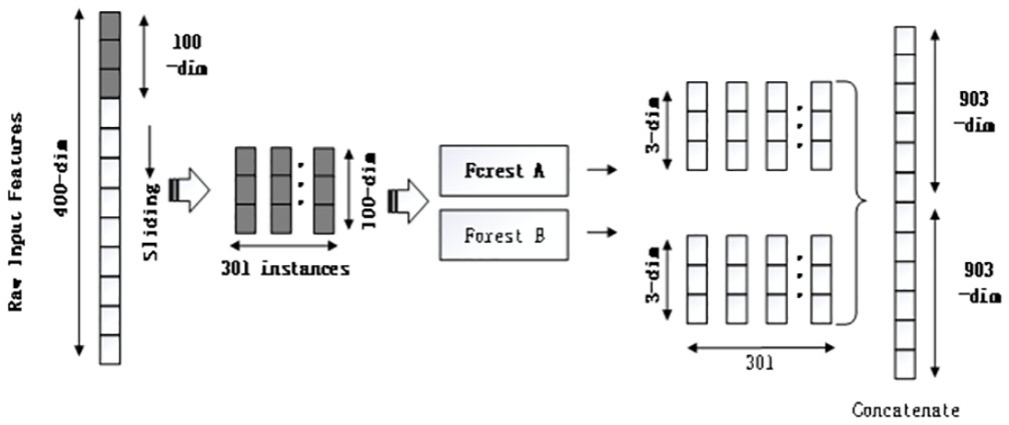
The framework of the multi-graining scanning.

The deep forest model divides the prediction process into two stages. One is the multi-granularity scanning stage. The other is the cascading forest stage. In the multi-granularity scanning stage, new samples are obtained using windows of different sizes. The output is a set of feature vectors generated by sliding the window. As shown in Fig 2, assuming that the original feature is 400 dimensions and the window size is 100, 301 new samples with 100 as the feature vector will be input into the forest. Unlike the convolutional layer operation of neural networks, the sliding window only scans the original features and does not involve parameters.

**Fig 3:**
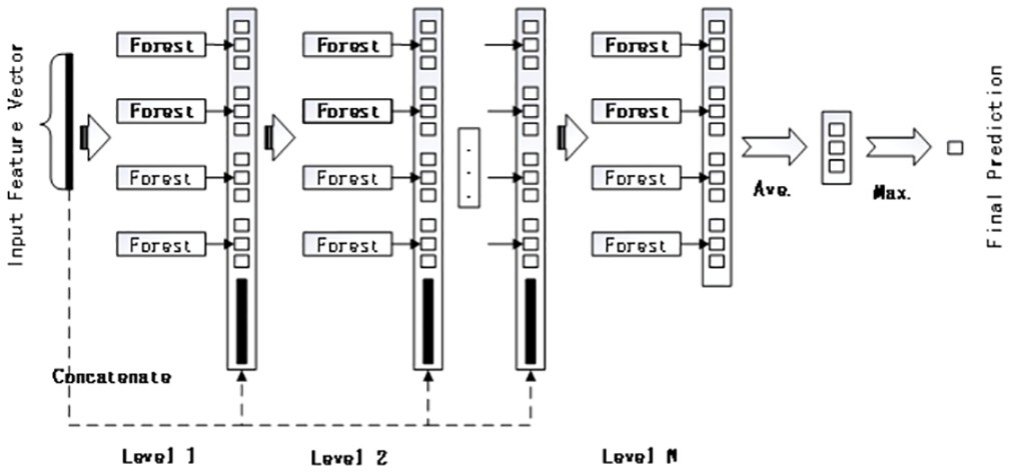
The framework of the cascade forest structure. Each cascade level consists of two random forests and two completely random forests.

Representation learning in deep neural networks mainly relies on the layer-by-layer processing of original features. Inspired by this recognition, deep forest adopts a cascading forest structure. As shown in Fig 3, each layer of the cascading forest stage comprises a forest composed of decision trees, two random forest models, and two complete random forest models. Each cascade layer receives the characteristic information processed by its previous level and outputs its processing results to the next level. According to the obtained feature representations, we can classify the input samples to get the final prediction result at the last level.

Multiple structures are vital for integrated learning [34, 35]. In general, the more complex the basic classifier, the greater the diversity of the deep forest. Based on sufficient diversity and reasonable accuracy of the basic classifier, satisfactory prediction results can be obtained. Therefore, ET and XGBoost were used to improve the basic classifier of the deep forest model cascading forest layer.

### 2.2 Cascade layer

#### 1)Extremely randomized trees

Extremely randomized Trees(ET) is a powerful classification method developed by Geurts [37], which has been widely used in various prediction problems [38–41]. Similar to the Random Forest, ET is integrated by many decision trees. It can effectively process high-dimensional data even without applying feature selection and has excellent classification effects. The difference is that the Random Forest finds the best bifurcation attribute in a random subset based on information gain. However, ET is an entirely random bifurcation value. It does not choose the optimal resolution threshold or feature, which reduces the variation of the model. Besides, Random Forest applies the Bagging model and uses bootstrap sampling to generate the training set. While ET uses all the training samples to obtain each decision tree, each decision tree applies all the same training samples. Since the ET training data set is all OOB (out-of-bag) data samples, the calculation of prediction error is the error calculation for OOB samples. Therefore, ET is superior to the Random Forest algorithm in terms of both the accuracy and the ability to fit the training data set [42].

#### 2)XGBoost

XGBoost is an efficient classification algorithm with a scalable machine learning system in a gradient lifting framework. This novel tree learning algorithm can process sparse data, due to its scalability and has a faster learning speed. In the experiment, XGBoost is more than ten times that of general machine learning algorithms [43]. In addition to parallel and distributed computing, XGBoost leverages off-core computing to scale to end-to-end systems of larger data with minimal clustered resources [44].

XGBoost is an integration of multiple decision trees. As shown in Eqs (1), (2), (3) and (4), the tree is 0 initially, and then the tree is added one by one based on the original. Each additional tree hopes that the effect can be improved.

$$\begin{array}{c} y_{i}^{0}=0 \end{array}$$ (1)

$$\begin{array}{c} y_{i}^{1}=f_{1}\left(x_{i}\right)=y_{i}^{0}+f_{1}\left(x_{i}\right) \end{array}$$ (2)

$$\begin{array}{c} y_{i}^{2}=f_{1}\left(x_{i}\right)+f_{2}\left(x_{i}\right)=y_{i}^{1}+f_{2}\left(x_{i}\right) \end{array}$$ (3)

$$\begin{array}{c} y_{i}^{t}=\sum_{k=1}^{t}f_{k}\left(x_{k}\right)=y_{i}^{t-1}+f_{t}\left(x_{i}\right) \end{array}$$ (4)

Here we need to ensure that adding new trees will improve the overall presentation, which means the objective function’s value (the loss) will decrease. Besides, if there are too many leaf nodes, overfitting is easy to occur. Therefore, an omega (ft) must be added to limit the number of leaf nodes. The omega (ft) formula is shown in Eq (5):

$$\begin{array}{c} \Omega \left(f_{t}\right)=\gamma T+\frac{1}{2}\gamma \sum_{j=1}^{T}w_{j}^{2} \end{array}$$ (5)

When looking for the best forking attribute, XGBoost optimizes inefficient greedy method. The idea is to use the percentile method to enumerate several possible attributes of the segmentation point and then find the best segmentation point. The complete objective function of the XGBoost algorithm is shown in Eq (6), which is composed of its loss function and regularized penalty term terms.

$$\begin{array}{c} {\text{Obj}}^{t}=\sum_{i=1}^{n}l(y_{i},y_{i}^{t})+\sum_{i=1}^{t}\Omega \left(f_{t}\right) \end{array}$$ (6)

### 2.3 Improved deep forest

**Fig 4:**
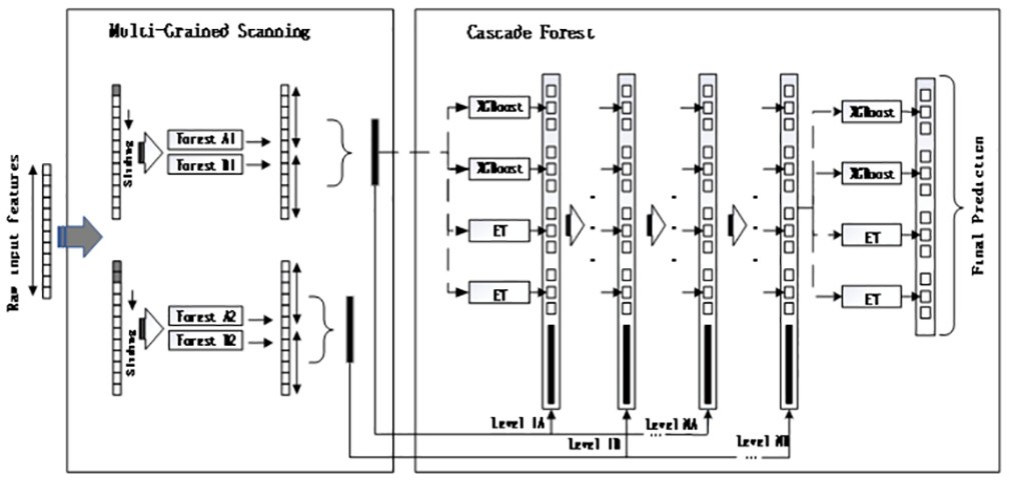
The framework of the improved deep forest.

The deep forest framework we used is shown Fig 4. Since the repurchase behavior of e-commerce consumers is predicted into two categories, we use two windows of different sizes to conduct the original features at the Multi—Grained Scanning. The two data sets obtained by scanning act on a Random Forest and a complete Random Forest, respectively, and converted into two feature vectors. The transformed feature vectors are then inputted into the second and third levels of the Cascade Forest stage for training. This process keeps repeating until performance convergence is verified. At the last level, four 3D classification vectors are aggregated, and the method with the maximum aggregation value is adopted to obtain the final classification result.

## 3. Experimental designs

Here is the simulation prediction environment. The operating system is Window10, the CPU is Intel (R) Core(TM) i5-8250u, the central frequency is 1.8 GHz, the memory is 8GB, and the simulation software is PYTHON3.7.

### 3.1 Feature engineering

Feature engineering is used to transform the raw data into feature data that can be directly used by the prediction model, which determines the upper limit of the machine semester. The training model is only as close to the upper limit as possible. So it is essential to construct a feature model in combination with specific problems.

This paper’s data come from the mobile e-commerce platform of Alibaba Tianchi competition. It is 2084859 consumer historical purchase behavior data from November 18 to December 18, involving 422,858 products and 1,054 categories. The data set is composed of a product information table and a user information table, and part of the content is shown in Tables 1 and 2. In order to protect the privacy of platform users, the relevant user and product information in the research uses numbers or letters to replace real information, where NaN means missing information.

**Table 1:** The first five rows of item information data.

Item_id	Item_geography	Item_category
100002303	NaN	3368
100003592	NaN	7995
100006838	NaN	12630
100008089	NaN	7791
100012750	NaN	9614

**Table 2:** The first five rows of user information data.

User_id	Item_id	Behavior_type	User_geography	Item_category	time
10001082	285259775	1	97lk14c	4076	2014-12-08 18
10001082	4368907	1	NaN	5503	2014-12-12 12
10001082	4368907	1	NaN	5503	2014-12-12 12
10001082	53616768	1	NaN	9762	2014-12-02 15
10001082	151466952	1	NaN	5232	2014-12-12 11

Table 1 contains three fields: product name, product location, and product type. Among them, the product location information is largely missing, and the product location information in the first five rows of the table is missing. After sorting out the data, it is found that among the 620,918 pieces of data, the product location information data is only 203410, which is about 1/3 of the total data. Therefore, product location information is not considered in subsequent experiments.

Table 2 contains six fields: user name, product name, user behavior, user location, product type, and behavior time. Similar to the product information data table, there is a large lack of user location information, which should not be used as one of the features in subsequent experiments. The user behavior in the table includes four types: click, favorite, add to cart, and purchase, which are represented by numbers 1, 2, 3, and 4.

**Fig 5:**
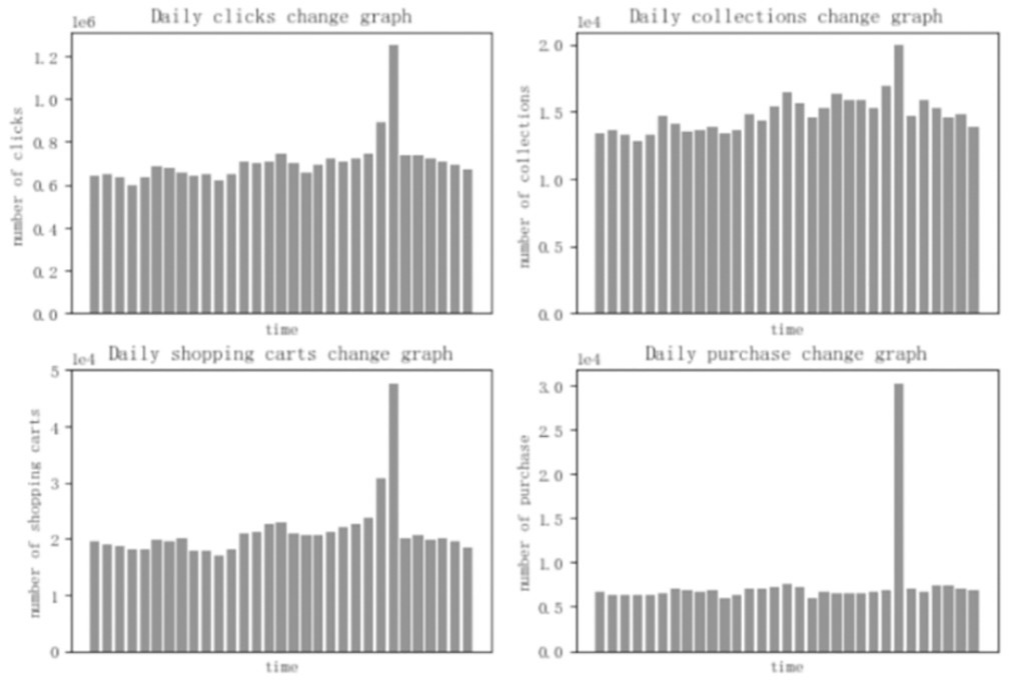
Daily user behavior data change graph.

Fig 5 is the daily historical behavior data of e-commerce consumers from November 18 to December 18, including four behaviors: click, bookmark, add to shopping cart, and purchase. As can be seen in the figure, due to the influence of promotional activities on Double Twelfth Day, the data has changed a lot and cannot represent the true behavior of consumers. Therefore, the behavior data from December 10 to December 12 is excluded.

After cleaning and combining the data, it is known that there are 232,379 items of consumer purchase behavior data and 20,673 items of consumer repurchase behavior data. Thus, it can be seen that only a small number of consumers will repurchase, resulting in an extreme imbalance between the data of repurchase behavior and the data of the non-repurchase behavior. Hence, we need to sample the data. Since repurchase behavior data is of great significance to the research results, they are all retained. The data of non-repurchase behaviors are kept randomly, making the quantity is twice as much as the data of repurchase behaviors.

The feature models of related researches are mainly divided into user features and product features refbib35,bib36,bib37. Given the question of whether e-commerce consumers will repurchase the products they have purchased, we eliminated the two factors of irrelevant users’ geographical location and behavior time. Then we sorted the left information into user characteristics and product characteristics. Since the consumers’ repurchase behavior connects users and products, the user-product interaction behavior features should be considered in the feature model. Therefore, we added three user interaction behavior factors. The first one is the total number of user clicks on a specific product. The second one is the total number of user collections of a specific product. The third one is the total number of adding to the shopping cart of a specific product by users. Finally, 13 influencing factors were determined for prediction experiments. Table 3 describes these factors in detail.

**Table 3:** Perdictive features of repurchase behavior of E—Commerce consumer.

Type	Feature	Name	Brief description
User characteristics	F1	User ID	Uniquely indicates user identity.
	F2	User clicks	The total number of times the user has clicked items.
	F3	User collects	The total number of times the user has collected items.
	F4	User adds	The total number of times users have added products to the shopping cart.
	F5	User purchases	The total number of times the user has purchased items.
Product characteristics	F6	Item ID	Uniquely indicates item identity.
	F7	Item category	Indicates the category of the product.
	F8	Click to buy rate	The ratio of the number of times the product was purchased to the number of times it was clicked.
	F9	Collect to buy rate	The ratio of the number of times the product was purchased to the number of times it was collected.
	F10	Add to buy rate	The ratio of the number of times the product was purchased to the number of times it has been added to the shopping cart.
User and commodity interaction behavior characteristics	F11	User clicks on the product	The number of times the user clicked the target product.
	F12	User’s collection of the product	The number of times the user collected the target product.
	F13	User’s addition of the product	The number of times the target product was added to the shopping cart by the user.

### 3.2 Evaluation criteria

According to the combination of the real category of the sample and the predicted category of the model in the paper, the results can be divided into four types: true positive (TP), false-positive (FP), true negative (TN), and false-negative (FN). The larger the value on the main diagonal is, the smaller the value on the sub-diagonal is, the better the model. After digitizing the confusion matrix, it is Precision, Recall, and F1-score. For the prediction problem of e-commerce consumers’ repurchase behavior, the accuracy and AUC value are added to evaluate the training and test results of the model. The specific formula is as follows:

Precision, the ratio of the sum of true positive (TP) to true positive (TP) and false-positive cases (FP):

$$\begin{array}{c} Precision=\frac{TP}{TP+FP} \end{array}$$ (7)

Recall the ratio of the sum of true positive (TP) to true positive (TP) and false-negative cases (FN):

$$\begin{array}{c} Recall=\frac{TP}{TP+FN} \end{array}$$ (8)

F1-score is the overall evaluation and weighted average based on precision and recall. “1” represents the best score of “F1”, while “0” represents the worst score. The formula is as follows:

$$\begin{array}{c} F1=\frac{2\times Precision\times Recall}{Precision+Recall} \end{array}$$ (9)

Accuracy, the ratio of the number of correctly classified samples to the total number of samples. The standard cannot reflect the potential distribution of response value, nor can it output the classifier’s error types. Nevertheless, it is easier to understand. The formula is as follows:

$$\begin{array}{c} Accuracy=\frac{TP+tn}{\left(TP+FP)+(TN+FN\right)} \end{array}$$ (10)

AUC (Area under the curve) is an evaluation indicator used for the binary classification model, indicating that a positive sample and a negative sample are randomly selected. The classifier can correctly give the probability that the score of the positive sample is higher than the negative sample, the area under the ROC curve. The larger the area, the better the model effect is, and the formula is as follows:

$$\begin{array}{c} AUC=\frac{\int_{0}^{1}TPdFP}{\left(TP+FN)(TN+FP\right)} \end{array}$$ (11)

### 3.3 Model comparison and parameter setting

To verify the effectiveness of feature engineering, we compared the experiment after adding the user-product interactive behavior characteristics with the experiment before adding them. To verify the performance of the improved deep forest, we compared the model with four other traditional machine learning models, including Linear regression (LR), K-nearest neighbor algorithm (KNN), Random Forest(RF), and Convolutional Neural Network(CNN). The parameter settings are shown in Table 4:

**Table 4:** Perdictive model parameter settings.

Model category	Main parameters	Value
LR	n_jobs	1
	Class_weight	balanced
	n_neighbors	5
KNN	leaf_size	30
	n_jobs	1
SVM	Kernel	Poly
	Gamma	10
	C	1
RF	n_estimators	10
	max_depth	10
	n_jobs	-1
CNN	learning_rate	0.001
	Number of convolution kernels	32
	Convolution kernel size	5*5
	Pooling core size	2*2
	Hidden layer neuron	128
	Number of epoch	100
	Optimization function	Tanh
	Activation function	ReLU

The parameter settings of the improved deep forest’s cascade layer are shown in Table 5:

**Table 5:** The cascade layer of improved deep forest parameters settings.

Cascade layer	Main parameters	Value
ET	n_estimators	10
	max_depth	10
	n_jobs	-1
XGBoost	n_estimators	10
	max_depth	10
	n_thread	0
	learning_rate	0.5

Besides, K-fold cross-validation was adopted to ensure the experimental results’ accuracy and objectivity, which means repeating the experiment K times. First, the original data were randomly divided into K data sets. Each experiment selected a different set from these K data sets as the test set, and the remaining K-1 is used as the training set for the experiment. Finally, the average value of the obtained K experimental results was taken. This method can avoid underfitting and overfitting and explain the model results better. In this case, let K be 10.

## 4. Result and analysis

Two experiments were used to analyze the feature model’s validity and the final prediction results’ effectiveness.

### 4.1 Feature model comparison

The prediction framework’s performance is verified by comparing the experimental results with and without adding user-product interaction behavior characteristics. The specific steps are: 1) Select all the balanced data, based on existing research, only use ten classic features including user characteristics and product characteristics for prediction; 2) Add three characteristics representing the interaction between users and products, and compare the classification results of this prediction model under the different number of features; 3) Verify the effectiveness of the feature model proposed in this research.

**Fig 6:**
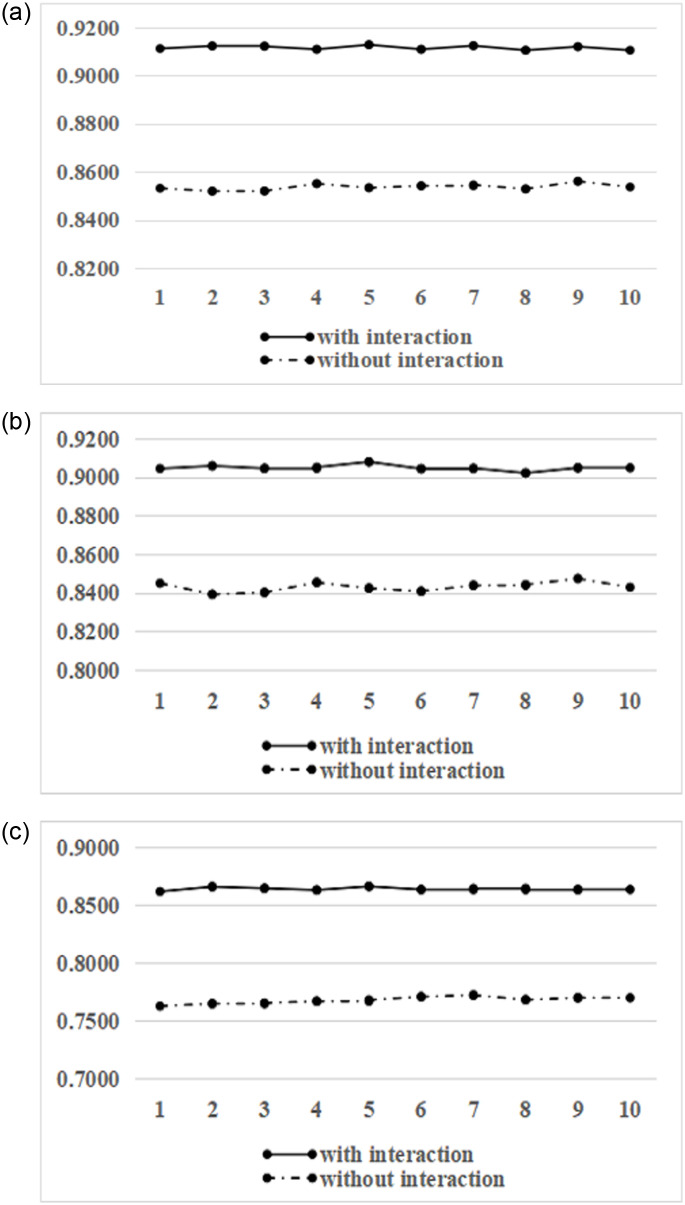
Comparison results under the different number of features. (a) Accuracy comparison chart. (b) AUC comparison chart. (c) FI-value comparison chart.

A total of 10 rows of model performance data were output through comparative analysis in feature model comparison. A line graph of the predicted results was drawn using the training grounds as the abscissa and the model performance as the ordinate. Fig 6(a)–6(c) respectively show the changing trend of accuracy, AUC, and F1-value. It can be seen that the feature model prediction after adding the user-product interaction behavior feature has been significantly improved. Moreover, the accuracy, AUC, and F1-value have been improved by 6.78%,7.31%,12.52%, respectively. It shows that the characteristics of user-product interaction behavior can improve the recognition degree of e-commerce consumers’ repurchase behavior by the feature model.

As far as this case is concerned, our prediction model’s feature selection method is reasonable and conforms to the e-commerce consumption system’s characteristics. However, whether it has advantages over the traditional prediction method needs to be further verified by the second experiment.

### 4.2 Prediction models comparison

By comparing the five prediction models of LR, KNN, SVM, RF, and CNN, the improved deep forest’s effectiveness is verified. All models are implemented through Python programming.

From the experimental results in Table 6, it can be seen that the accuracy of LR is low, only 0.6626, indicating that consumer behavior data presents a linearly indivisible state. SVM introduces a kernel function suitable for nonlinear data. Compared with LR, the accuracy is improved by 0.2201. However, the recall rate decreased by 0.229, which caused the F1-value of SVM to decrease by 0.0544, which made the result unsatisfactory. The accuracy of KNN is 0.1515 higher than LR and 0.0586 lower than SVM, but the recall rate is relatively high, so F1-value is higher than LR and SVM. Compared with the previous three prediction models, RF has good accuracy and recall, so F1-value is increased to 0.8306. The accuracy of CNN is as high as 0.8891, but the recall rate is sacrificed like SVM, resulting in F1-value not optimal. In terms of accuracy, gcForest is slightly lower than CNN, but the recall rate is increased by 5.05%, so F1-value is higher than CNN, an increase of 2.44%. Under comprehensive consideration, the improved deep forest has the best behavior prediction performance of e-commerce consumers.

**Table 6:** Performance comparison of predictive models.

Model	Precision	Recall	F1-score
LR	0.6626	0.7333	0.6961
KNN	0.8141	0.6104	0.6976
SVM	0.8827	0.5043	0.6417
RF	0.8824	0.7845	0.8306
CNN	0.8891	0.8016	0.843
Improved DF	0.8862	0.8421	0.8636

**Fig 7:**
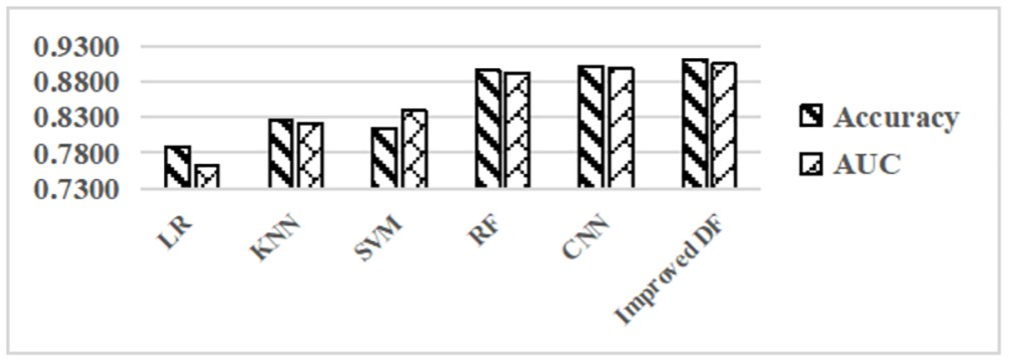
Performance comparison of predictive models.

Fig 7 compares the prediction effect of the model in terms of accuracy and AUC. From left to right, the accuracy and AUC of each model show a stepwise increase except that SVM’s accuracy is lower than KNN. The accuracy and AUC of the improved deep forest are higher than other prediction models.

**Fig 8:**
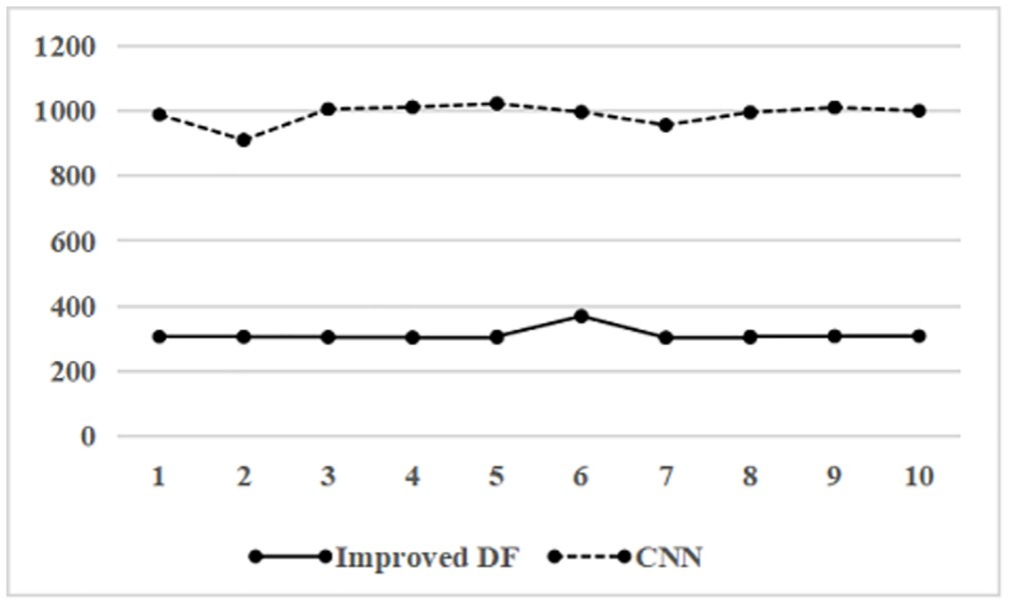
Comparison of training time between deep forest and CNN.

To verify the time cost of the improved deep forest more intuitively, the training time was compared with CNN, and the result was shown in Fig 8. The CNN’s training time and the improved deep forest are relatively stable and fluctuate slightly within a fixed interval. During the experiment, the feature columns, which were sorted in the memory as blocks, were not reused in iterations. Although the iterations in the improved deep forest must be serialized, each feature column can be processed in parallel, effectively increasing the model’s efficiency. Therefore, the improved deep forest has a short training time, almost 1/3 that of CNN, which has obvious cost advantages.

Based on the above experiments, we can know that improved deep forest has good prediction performance. Compared with CNN, accuracy increased by 1.15%, AUC increased by 0.7%, F1-value increased by 2.44%, and training time decreased by 2.17 times. It can be seen that the improvements we have made based on the deep forest algorithm can improve the prediction results of e-commerce consumers’ repurchase behavior and verify the effectiveness of the model.

## 5. Discussion

Aiming at the prediction problem of e-commerce consumers’ repurchase behavior, we improved a prediction model based on deep forest. Through verification experiments, we discussed the research results of the improved deep forest model:

- The new feature engineering program, after adding the characteristics of user-product interactive behavior, has a better performance in predicting and analyzing consumer behavior. The accuracy, AUC, and F1-value have been increased by 6.78%, 7.31%, and 12.52%, respectively.
- Compared with other predictive models, the results of consumer repurchase behavior obtained by improved deep forest have higher accuracy, recall, and AUC values. Compared with the neural network model, although the accuracy rate is slightly lower, the recall rate increases by 5.05%, and the F1-value increases by 2.44%. The feature dimension of this method is small, and each feature can be processed in parallel. Consequently, while ensuring the accuracy of the model results, the training time cost is reduced by 2/3.
- The experiment in this paper is based on the real consumption data of e-commerce, which helps the prediction model based on gcForest to adapt to the e-commerce industry. In addition, it can also be used to predict any e-commerce consumer who has a purchase history. And it can be used in the smart recommendation system of the e-commerce platform to help companies achieve low-cost precision marketing.

## 6. Conclusion

In this paper, we put forward an improved deep forest model to predict the repurchase behavior of e-commerce consumers. Compared with the existing repurchase behavior prediction model, our feature engineering considers the user characteristics and product characteristics and considers the characteristics of user-product interactive behavior. Also, by comparing with traditional machine learning models, the effectiveness of the improved deep forest in predicting the repurchase behavior of e-commerce consumers is verified. However, consumer behavior research is a complex issue, and the use of behavioral data alone is not enough to support the entire research. When constructing feature projects, more consideration can be given to differences in consumer personality characteristics, such as gender, age, consumption level, consumer psychology, etc., in order to obtain more valuable features. In the follow-up research, it’s meaningful to build a predictive model that includes individual user differences from the level of the influence mechanism and then provide more valuable suggestions for increasing e-commerce consumers’ repurchase behavior.

## References

[1] Chen X W, Lin X. Big Data Deep Learning: Challenges and Perspectives. IEEE Access, 2014, 2(2), 514–525. doi: 10.1109/ACCESS.2014.2325029

[2] Goel S, Hofman J M, Lahaie S. Predicting consumer behavior with Web search. Proceedings of the National Academy of Sciences of the United States of America. 2010, 107(41), 17486–17490. doi: 10.1073/pnas.1005962107 20876140 PMC2955127

[3] Koehn D, Lessmann S, Schaal M. Predicting Online Shopping Behaviour from Clickstream Data using Deep Learning. Expert Systems with Applications. 2020, 150, 113342. doi: 10.1016/j.eswa.2020.113342

[4] Cheng Y, Liu Z. China Internet Network Information Center. The 46th China Statistical Report on Internet Development. 2020, 7(2), 36–38.

[5] Victor Mayer-Schonberger, Kenneth Kukje, Viktor Mayer-Schonberger. The era of big data: a big change in life, work, and thinking on Zhejiang 2013, 20–22.

[6] Obermeyer Z, EmanuelE E J. Predicting the Future—Big Data, Machine Learning, and Clinical Medicine. N Engl J Med. 2016, 375(13), 1216–1219. 27682033 10.1056/NEJMp1606181 PMC5070532

[7] Xindong W, Gong-Qing W, Xingquan Z, et al Data mining with big data. IEEE Transactions on Knowledge and Data Engineering. 2014, 26(1), 97–107. doi: 10.1109/TKDE.2013.109

[8] Chen H, Chiang R H L, Storey V C. Business Intelligence and Analytics: From Big Data to Big Impact. Society for Information Management and The Management Information Systems Research Center. 2012, 36(4), 1165–1188.

[9] Nadeem M A, Liu Z, Pitafi H, et al. Investigating the repurchase intention of Bitcoin: empirical evidence from China. Data Technologies and Applications. Data Technologies and Applications. 2020, 54(5), 625–642. doi: 10.1108/DTA-10-2019-0182

[10] Prodanova J, Sonia San-Martín, Nadia Jiménez. Achieving customers’ repurchase intention through stimuli and site attachment. Journal of Organizational Computing and Electronic Commerce. 2020, 30(3), 1–22. doi: 10.1080/10919392.2020.1739395

[11] Wang Y, Anderson J, Joo S J, et al. The leniency of return policy and consumers’ repurchase intention in online retailing”. Industrial Management and Data Systems. 2019, 120(1), 21–39. doi: 10.1108/IMDS-01-2019-0016

[12] Lzroiu G, Popescu G H, Nica E. The role of electronic word-of-mouth in influencing consumer repurchase intention in social commerce. SHS Web of Conferences. 2020, 74(2), 1209–1238.

[13] Zerbini C, Vergura D T, Latusi. A new model to predict consumers’ willingness to buy fair-trade products. Food Research International. 2019, 122, 167–173. doi: 10.1016/j.foodres.2019.04.008 31229069

[14] Xu Y, Zhang W, Bao H, et al. A SEM–Neural Network Approach to Predict Customers’ Intention to Purchase Battery Electric Vehicles in China’s Zhejiang Province. Sustainability. 2019, 11(11), 1–19. doi: 10.3390/su11113164

[15] Verma V K, Chandra B. An application of theory of planned behavior to predict young Indian consumers’ green hotel visit intention. Journal of Cleaner Production. 2017, 172(1), 52–1162.

[16] Liu Cheng-Ju, Huang Tien-Shou, Ho Ping-Tsan. Machine learning-based e-commerce platform repurchase customer prediction model. Plos One. 2020, 15(12). doi: 10.1371/journal.pone.0243105 33270714 PMC7714352

[17] Kumar Anil, Kabra Gaurav, Mussada Eswara Krishna. Combined artificial bee colony algorithm and machine learning techniques for prediction of online consumer repurchase intention. Neural computing and Applications. 2019, 31(2), 877–890.

[18] Chiu C, Shu C H. Monthly car sales prediction using Internet Word-of-Mouth (eWOM). IEEE International Conference on Innovations in Intelligent Systems and Applications on Gdynia POLAND. 2017, 345–348.

[19] Bhatti U A, Yuan L, Yu Z, et al. Predictive Data Modeling Using sp-kNN for Risk Factor Evaluation in Urban Demographical Healthcare Data. Journal of Medical Imaging and Health Informatics. 2021, 11(1), 7–14. doi: 10.1166/jmihi.2021.3313

[20] Sanjar K, Bekhzod O, Kim J. Missing Data Imputation for Geolocation-based Price Prediction Using KNN MCF Method. International Journal of Geo-Information. 2020, 9(4), 227 doi: 10.3390/ijgi9040227

[21] Yin Z, Hou J. Recent advances on SVM based fault diagnosis and process monitoring in complicated industrial processes. Neurocomputing. 2016, 174(22), 643–650. doi: 10.1016/j.neucom.2015.09.081

[22] Cherkassky V. The nature of statistical learning theory. IEEE Trans. Neural Netw. 1997, 8(6), 1564. doi: 10.1109/TNN.1997.641482 18255760

[23] Vapnik V.N., Vapnik V. Statistical Learning Theory on Wiley New York. 1998, 123–125.

[24] Chen W, Li Z, Zhang M. Linear and Non-Linear Models for Purchase Prediction”. the 2015 International ACM Recommender Systems Challenge on ACM 1–4.

[25] AZW, CCLAB, CXCB, et al. Flood hazard risk assessment model based on random forest. Journal of Hydrology. 2015, 527, 1130–1141. doi: 10.1016/j.jhydrol.2015.06.008

[26] Goel S, Hofman J M, Lahaie S. Forecasting tourism demand to Catalonia: Neural networks vs. time series models—ScienceDirect. Economic Modelling. 2014, 36(1), 220–228.

[27] Tin-Shyug Lee, Chih-Chou Chiu, Chou Yuchao. Mining the Customer Credit Using Classification and Regression Tree and Multivariate Adaptive Regression Splines. Computational Statistics and Data Analysis. 2006, 50(4), 1113–1130. doi: 10.1016/j.csda.2004.11.006

[28] Torra C S. Forecasting tourism demand to Catalonia: Neural networks vs. time series models. Economic Modelling. 2014, 36, 220–228. doi: 10.1016/j.econmod.2013.09.024

[29] Ahmad M W, Mourshed M, Rezgui Y. Trees vs Neurons: Comparison between random forest and ANN for high-resolution prediction of building energy consumption. Energy and Buildings. 2017, 147,77–87. doi: 10.1016/j.enbuild.2017.04.038

[30] Zhou Z H, Feng J. Deep Forest: Towards An Alternative to Deep Neural Networks. Twenty-Sixth International Joint Conference on Artificial Intelligence. 2017, 3353–3357.

[31] Chica-Olmo M, Chica-Rivas. Machine learning predictive models for mineral prospectivity: An evaluation of neural networks, random forest, regression trees and support vector machines Ore Geology Reviews Journal for Comprehensive Studies of Ore Genesis and Ore Exploration. 2015, 71, 804–818.

[32] Hou C, Chen C, Wang J. Tree-Based Feature Transformation for Purchase Behavior Prediction. IEICE Transactions on Information and Systems. 2018, 101(5), 1441–1444. doi: 10.1587/transinf.2017EDL8210

[33] Hamsa H, Indiradevil S. Student academic performance prediction model using decision tree and fuzzy genetic algorithm. 1st Global Colloquium on Recent Advancements and Effectual Researches in Engineering 2016,326–332.

[34] Ma C, Liu Z, Cao Z, et al. Cost-Sensitive Deep Forest for Price Prediction. Pattern Recognition. 2020, 107. doi: 10.1016/j.patcog.2020.107499

[35] Muhammad Usman Riaz, Luo Xiao Guang, et al. Consumers’ purchase intention and decision-making process through social networking sites: a social commerce construct. Behaviour and Information Technology. 2021, 40, 99–115. doi: 10.1080/0144929X.2020.1846790

[36] Bratu S. Can social media influencers shape corporate brand reputation? online followers’ trust, value creation, and purchase intentions. Review of Contemporary Philosophy, 2019, 18.

[37] Geurts P, Ernst D, Wehenkel L. Extremely randomized trees. Mach Learn. 2016, 63, 3–42. doi: 10.1007/s10994-006-6226-1

[38] Basith S, Manavalan B, Shin TH, Lee G. iGHBP: computational identification of growth hormone binding proteins from sequences using extremely randomised tree. Comput Struct Biotechnol J. 2018, 16(4), 12–20. doi: 10.1016/j.csbj.2018.10.007 30425802 PMC6222285

[39] Alswaina F, Elleithy K. Android Malware Permission-Based Multi-Class Classification Using Extremely Randomized Trees. IEEE Access. 2018, 2(1), 1–1.

[40] Xia B, Zhang H, Li Q.M, Li T. PETs: A Stable and Accurate Predictor of Protein-Protein Interacting Site Based on Extremely Randomized Trees. IEEE Trans. Nanobiosci. 2015, 14, 882–893. doi: 10.1109/TNB.2015.2491303 26529772

[41] Manavalan B, Basith S, Shin T H, et al. AtbPpred: A Robust Sequence-Based Prediction of Anti-Tubercular Peptides Using Extremely Randomized Trees. Computational and Structural Biotechnology Journal. 2019, 17, 972–981. doi: 10.1016/j.csbj.2019.06.024 31372196 PMC6658830

[42] Uddin M.T.; Azher M.U. Human Activity Recognition from Wearable Sensors using Extremely Randomized Trees Human Activity Recognition from Wearable Sensors using Extremely Randomized Trees. Dhaka, Dhaka, Bangladesh, 2015.

[43] Song Peiyi, Liu Yutong. An XGBoost Algorithm for Predicting Purchasing Behaviour on E-Commerce Platforms. TEHNICKI VJESNIK-TECHNICAL GAZETTE. 2020, 27(5), 1467–1471.

[44] Chen Tianqi, Guestrin Carlos. XGBoost: A Scalable Tree Boosting System. 22nd ACM SIGKDD International Conference on Knowledge Discovery and Data Mining (KDD). 2015, 785–794.

